# Trends in genetic diversity for all Kennel Club registered pedigree dog breeds

**DOI:** 10.1186/s40575-015-0027-4

**Published:** 2015-09-21

**Authors:** T. W. Lewis, B.M. Abhayaratne, S. C. Blott

**Affiliations:** The Kennel Club, 1-5 Clarges Street, Piccadilly, London, W1J 8AB UK; School of Veterinary Medicine and Science, The University of Nottingham, Sutton Bonington Campus, Sutton Bonington, Leicestershire LE12 5RD UK

**Keywords:** Dogs, Population genetics, Inbreeding, Effective population size

## Abstract

**Background:**

Inbreeding is inevitable in closed populations with a finite number of ancestors and where there is selection. Therefore, management of the rate of inbreeding at sustainable levels is required to avoid the associated detrimental effects of inbreeding. Studies have shown some pedigree dog breeds to have high levels of inbreeding and a high burden of inherited disease unrelated to selection objectives, implying loss of genetic diversity may be a particular problem for pedigree dogs. Pedigree analysis of all 215 breeds currently recognised by the UK Kennel Club over the period 1980–2014 was undertaken to ascertain parameters describing the rate of loss of genetic diversity due to inbreeding, and the presence of any general trend across all breeds.

**Results:**

The trend over all breeds was for the rate of inbreeding to be highest in the 1980s and 1990s, tending to decline after 2000. The trend was comparable in very common and rarer breeds, although was more pronounced in rarer breeds. Rates of inbreeding over the entire period 1980–2014 were not correlated with census population size. The existence of popular sires was apparent in all breeds.

**Conclusion:**

The trends detected over 1980–2014 imply an initial excessive loss of genetic diversity which has latterly fallen to sustainable levels, even with modest restoration in some cases. The theory of genetic contributions, which demonstrates the fundamental relationship of inbreeding and selection, implies that popular sires are the major contributor to high rate of inbreeding.

**Electronic supplementary material:**

The online version of this article (doi:10.1186/s40575-015-0027-4) contains supplementary material, which is available to authorized users.

## Lay Summary

Inbreeding’ is widely viewed as being harmful to the wellbeing of individuals and populations. In populations of a limited size, complete avoidance of breeding between individuals with a shared ancestry quickly becomes impossible. Furthermore, selection within dog breeds for desirable traits will inevitably result in the breeding of individuals that resemble each other with respect to the traits under selection. The resemblance of relatives is a fundamental principle of genetics, and means that selected individuals will on average be more closely related than a random pair taken from across the population. Therefore some degree of inbreeding is inevitable in all populations; it is how quickly this changes that is informative. The rate of inbreeding in a population relates to the risk of detrimental effects associated with inbreeding (such as loss of genetic diversity, inbreeding depression and the spread of deleterious genetic variants). Therefore the rate of inbreeding is a measure of the sustainability of a population. This study reports the general trends in the rate of inbreeding observed through population analyses of all 215 pedigree dog breeds currently recognised by the UK Kennel Club, over the period 1980 to 2014.

For all breeds, the trend was for the rate of inbreeding to be highest in the 1980s and 1990s, representing a major contraction in genetic diversity. Since 2000 however, the general trend has been for the rate of inbreeding to decline to sustainable levels, with some modest restoration of genetic diversity in some cases. It is interesting that this coincides with the relaxation of the UK’s quarantine laws, and is possibly due to the more widespread use of non-UK animals for breeding. The rate of inbreeding (or effective population size) showed no relationship with the actual population size, as judged by mean number of KC registrations. Evidence of popular sires was common in all breeds. Popular sires make large genetic contributions to subsequent generations and are the biggest influence on the rate of inbreeding. There was variation among breeds in the trend of rate of inbreeding over the period 1980-2014. Reports detailing results of the population analysis for each individual breed are publically available at: http://www.thekennelclub.org.uk/vets-researchers/publications,-statistics-and-health-results/breedpopulation-analyses.

## Background

It is widely believed that pedigree dogs are very inbred, due to closed registries and breeding practices, and that this has had a detrimental effect on the health and welfare of many pedigree breeds. Indeed studies have empirically determined a large depletion in genetic diversity in some pedigree dogs breeds [1–3], and many breeds do suffer a high burden of genetic disease [4].

Inbreeding is unavoidable in finite populations, since the number of ancestors increases exponentially per generation (2^n^, where *n* is the generation, i.e. 2 parents, 4 grand-parents, 8 great-grand-parents, and so on). This very quickly leads to an unfeasible number of unrelated ancestors at the *n*^*th*^ generation, implying common ancestry and so inbreeding. Where there is common ancestry the probability that both alleles inherited by an individual are copies of a single allele from a common ancestor to both parents is >0. The coefficient of inbreeding (F) is this probability, and F for an individual will be higher if there are more common ancestors and in more recent generations. Given that all individuals carry mutant deleterious alleles, many of which when inherited in duplicate can result in loss of function, F describes the risk to an individual. A high level of inbreeding also has detrimental effects on the wider population, resulting in the loss of genetic variation, loss of heterozygosity, possible effects of inbreeding depression, and a higher rate of spread of deleterious alleles [5].

However, what is less widely understood is the fundamental relationship between inbreeding and selection. The rate of inbreeding (ΔF, how quickly F rises over generations) and genetic gain (the change in mean genetic liability across generations in response to selection) have been shown to be related by the theory of genetic contributions [6–8]. This intrinsic relationship means that a response to selection, elicited by a departure from random mating, cannot be achieved without concomitant ΔF being >0, indicating some loss of genetic diversity. Therefore, given that avoidance of co-ancestry in finite populations is impossible and the necessity of selection to provide a widespread and lasting improvement in welfare where there is inherited disease, management of ΔF at sustainable levels is key.

While F may be considered to describe the risk to an individual from the detrimental effects of inbreeding, effective management of ΔF goes beyond simply seeking to minimise F of future offspring [5]. An apposite, although extreme, hypothetical example would be the repeated use of an unrelated sire to all females in a small breeding population. While the progeny of such matings would all have F = 0, they would all be half siblings, and the subsequent generation produced from them would all have F = 0.125. ΔF over the generation from progeny to grand-progeny of the unrelated sire would also be 0.125. This example also demonstrates how popular sires, those making a large genetic contribution to the population, have a major influence on ΔF.

ΔF is often expressed as the effective population size, N_e_ (see Methods), which is defined by Hill and Zhang [9] as “the size of an idealised population with the same increment in drift or inbreeding per generation [as is observed]”. The rate of loss of genetic diversity within a breed or population increases dramatically when N_e_ <100 [10], while a population with N_e_ <50 is considered to be at high risk of the detrimental effects of inbreeding [11]. Therefore, ΔF and N_e_ can be used to determine the history and sustainability of populations and inform appropriate breeding strategies where the aims are genetic improvement and the conservation of genetic resources.

The objective of this study was to report the results of population analyses determining genetic parameters (ΔF and N_e_) and population dynamics, such as number of registrations and the extent of popular sire usage, over the period 1980–2014 for all 215 breeds currently recognised by the UK Kennel Club. Statistics are reported for the whole period, and over seven 5-year blocks (1980–1984, 1985–1989, 1990–1994, 1995–1999, 2000–2004, 2005–2009, 2010–2014) to determine variations within an overall trend.

## Results

Reports of available results for all 215 breeds recognised by the Kennel Club are publically available at http://www.thekennelclub.org.uk/vets-researchers/publications,-statistics-and-health-results/breed-population-analyses. Where registrations for a breed have consistently been small (e.g. Komondor) or have recently risen from zero over the majority of the period 1980–2014 (e.g. Turkish Kangal Dog), partial reports are given.

### Overview

#### Registrations

While identification of the causes of large changes in the number of registrations in a breed over 1980–2014 is beyond the remit of this investigation, these unknown drivers of breed popularity may also have had an impact on breeding strategies and so may be of interest. A plot of number of registrations per year of birth over the period 1980–2014 is included in reports for all breeds. Notable breeds with large increases in popularity, as determined by number of registered animals per year of birth, include the Pug (regression coefficient of number registered on year = 194.8, s.e. 24.24), Dogue de Bordeaux (94.8, s.e. 9.44) and French Bulldog (127.4, s.e. 28.07); while those exhibiting large decreases in registrations include the Yorkshire Terrier (−381.8, s.e. 75.80), Rough Collie (−131.6, s.e. 12.70) and Dobermann (−149.8, s.e. 31.77). However, examination of individual breed plots of registrations on year of birth reveal distinct trends within the period 1980–2014 in many cases.

#### Sire usage

**Fig. 1:**
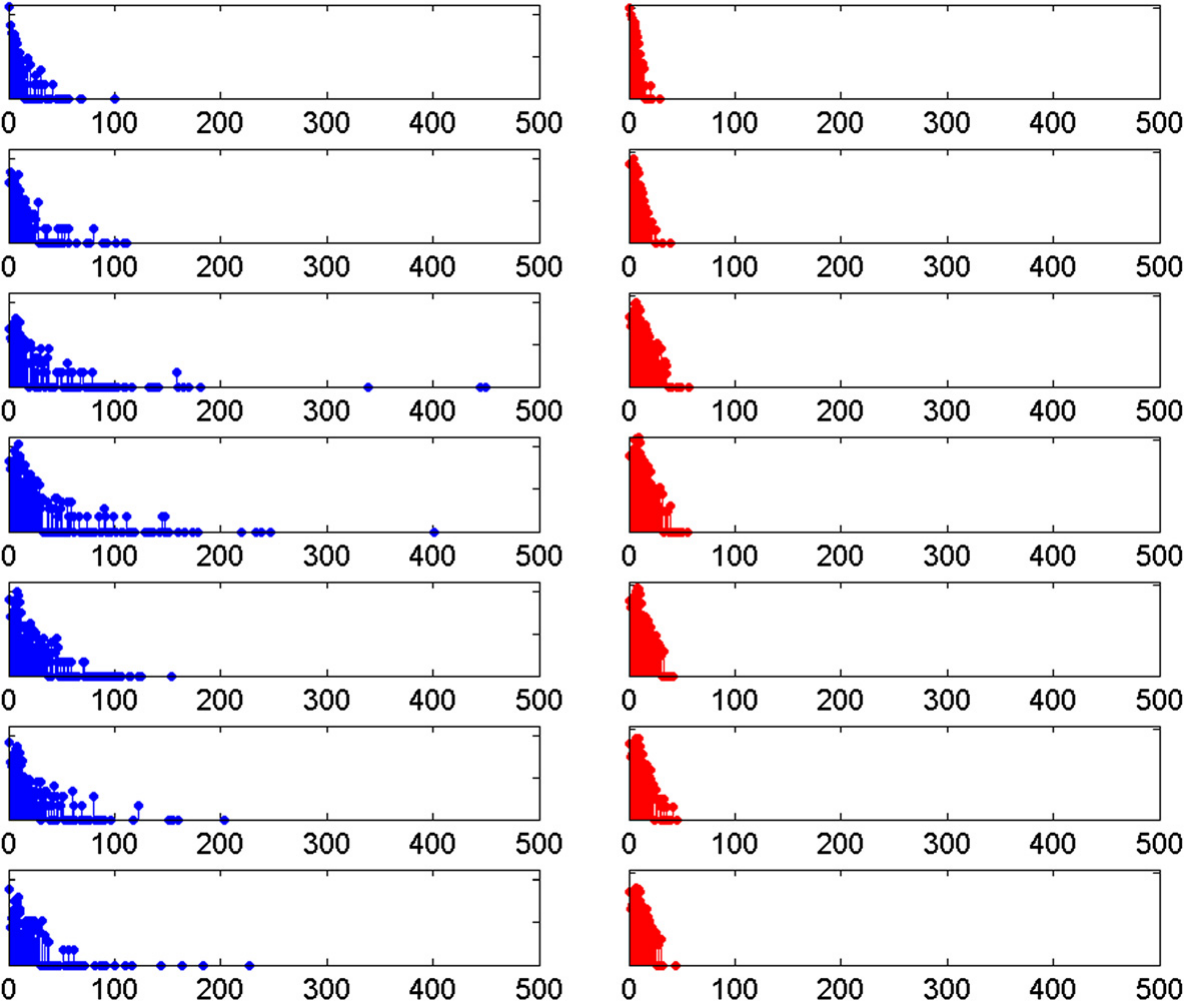
Histograms of progeny per sire and dam. Histograms of progeny per sire (blue) and per dam (red) for the Dalmatian breed in each of the seven 5 year blocks: 1980–1984 (top), 1985–1989, 1990–1994, 1995–1999, 2000–2004, 2005–2009, 2010–2014 (bottom)

The report for each breed indicates the total number of unique sires; the maximum, mean, median, mode and standard deviation in number of progeny per sire; and the percentage of registered animals born to the most prolific 50 %, 25 %, 10 % and 5 % of sires, per year. In all breeds (with sufficient data) there was evidence of ‘popular sires’, with more dams used than sires and the most prolific males siring a large proportion of puppies in a breed per year. This phenomenon occurs in virtually all domestic mammal species, due to the biological limitations of reproductive capacity of females compared to males, and results in the wider use of a smaller number of males. The disparity in numbers of males and females used for breeding has an impact of a higher selection intensity being applied to males. Histograms of progeny per sire and dam in each of the seven 5-year blocks provide a visual illustration of the extent of (and changes in) popular sire usage in individual breed reports (Fig. 1).

#### Generation Interval (L)

The generation interval is the mean age of parents at the birth of progeny which themselves go on to reproduce. For breeds with a mean of >50 registrations per annum (in each of the seven 5-year blocks, *n* = 121), the mean whole period generation interval ranged from 3.06 years (Miniature Bull Terrier) to 5.04 years (Bearded Collie), with a mean of 3.88 years and standard deviation of 0.412 years.

#### Annual mean observed and expected inbreeding coefficients

**Fig. 2:**
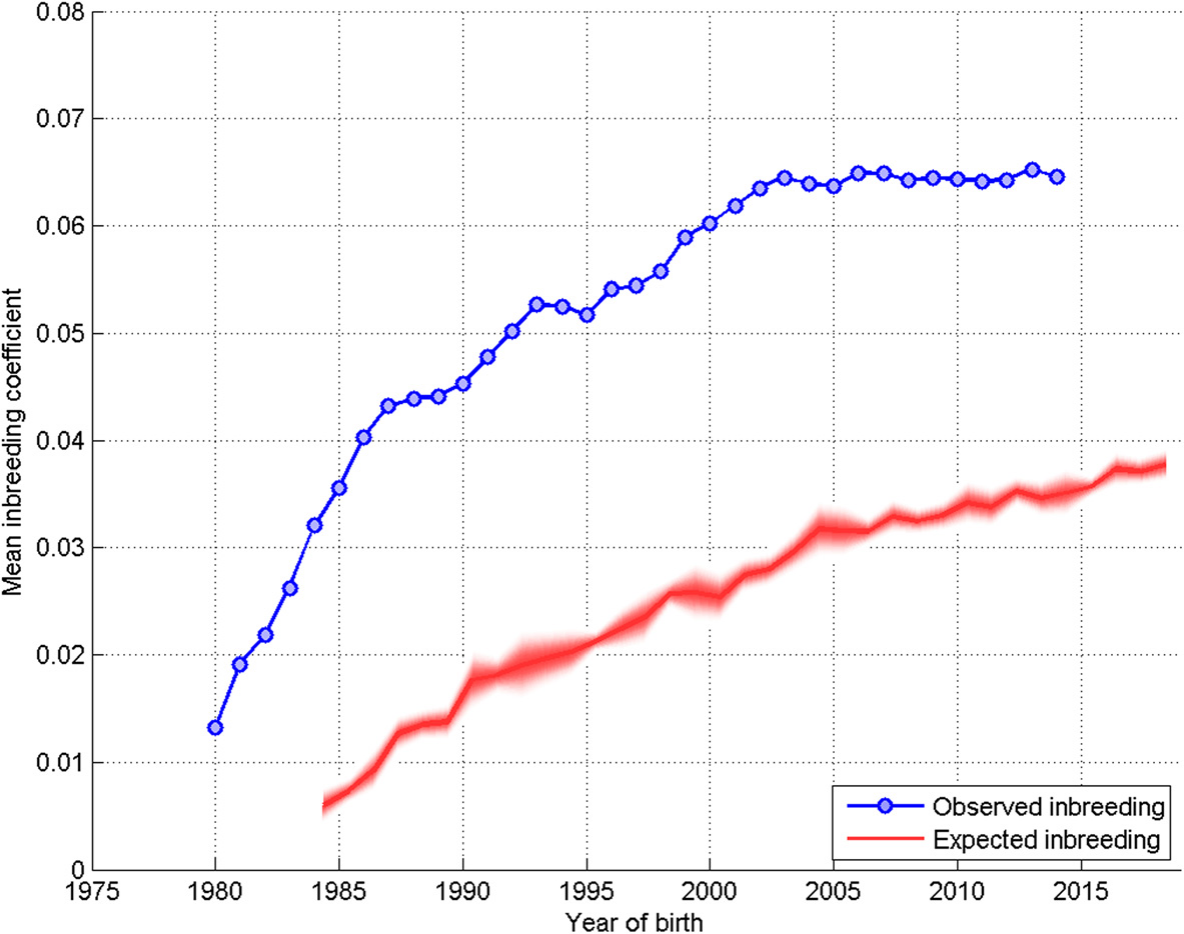
Observed and expected inbreeding coefficient. Plot of observed mean inbreeding coefficient (blue) and mean inbreeding coefficient expected from random mating (red) on year of birth for the Labrador Retriever breed. The ‘bleeding’ around the expected inbreeding line indicates the standard deviation of 10 sample mean kinship coefficients of 50 individuals (see methods)

Where sufficient data allow, breed reports include a plot of the mean inbreeding coefficient (observed inbreeding) and the [sample] mean of the coefficient of kinship (expected inbreeding [from random mating]) of all animals born per year. Expected inbreeding is staggered by the breed mean generation interval (*L*, since animals born in year *t* would on average produce breeding progeny at *t + L* in the future). The steepness of the ‘observed inbreeding’ line illustrates the rate of inbreeding and so rate of loss of genetic diversity; the steeper the gradient the more rapid the loss. Figure 2 is an example plot from the Labrador Retriever breed.

#### Rate of inbreeding (ΔF) and effective population size (N_e_)

**Fig. 3:**
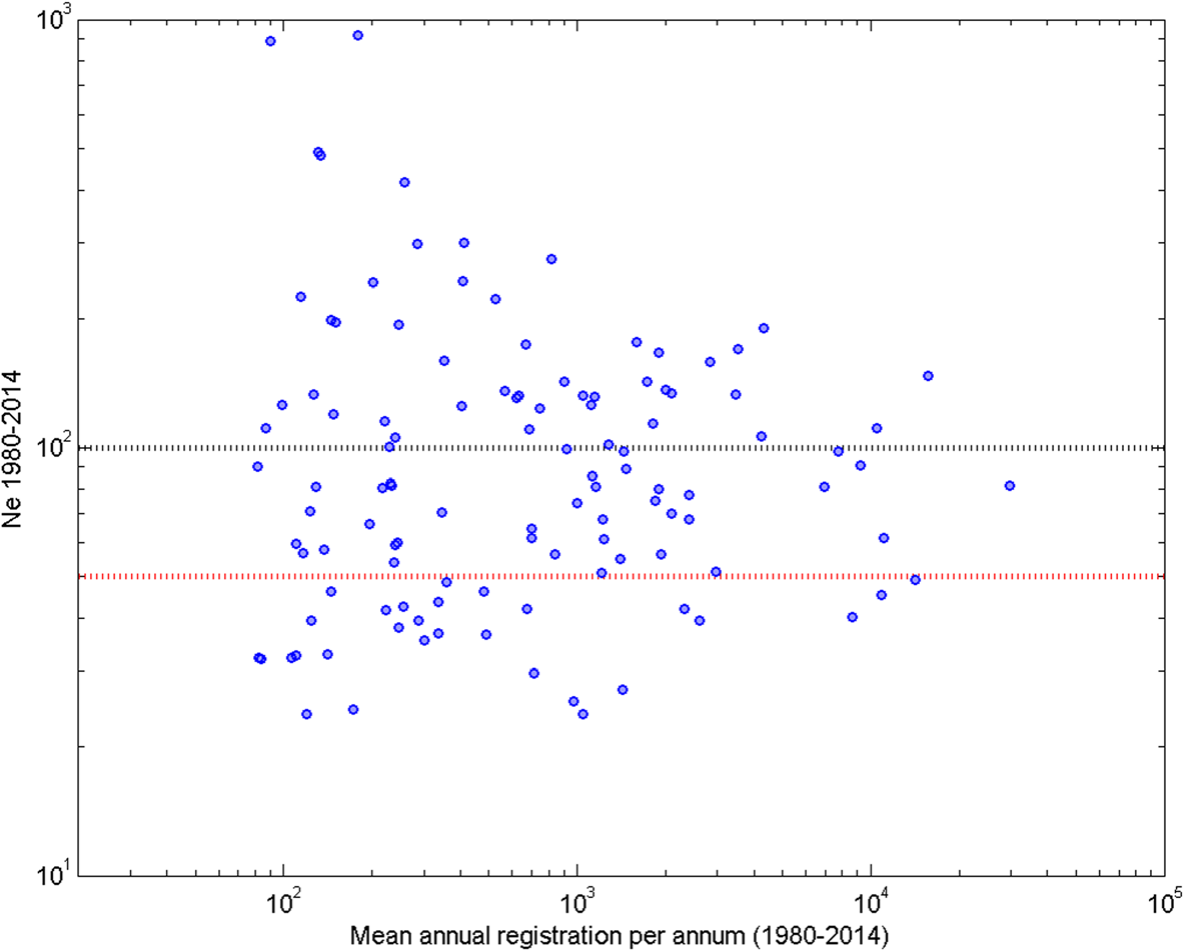
Scatter plot of effective population size on mean registrations per year. Scatter plot of whole period (1980–2014) effective population size (N_e_) on mean annual number of registrations for all 116 breeds with an average of >50 registrations in each of the seven 5-year blocks (1980–1984 to 2010–2014) and determinable N_e_. The black dashed line indicates N_e_ of 100, and the red dashed line N_e_ of 50. Note both axes are on a logarithmic scale

Of all breeds with an average of >50 registrations per annum (over each of the seven 5-year blocks, *n* = 121), five had a negative whole period ΔF implying an apparent overall increase in genetic diversity, and consequently no determinable N_e_ (Bernese Mountain Dog, Briard, Standard Poodle, Rhodesian Ridgeback and Tibetan Terrier). Of the 116 remaining breeds, the N_e_ calculated over the period 1980–2014 ranged from 23.8 (Manchester Terrier) to 918.8 (Borzoi). Of these 116 breeds, 68 had N_e_ of <100, with 29 having N_e_ of <50. A scatter plot of N_e_ calculated over the period 1980–2014 on mean annual registrations over the same period for the 116 breeds where N_e_ was determinable is shown in Fig. 3. There was no statistically significant association between N_e_ and census population size as measured by mean registrations.

A table detailing the whole period rate of inbreeding per annum, *L* and N_e_ for the 121 breeds with an average of >50 registrations per annum over each of the seven 5-year blocks is shown in Additional file 1. The same statistics, where meaningful due to sufficient data, and the average number of registrations per year over each of the seven 5-year blocks for the remaining 94 breeds is shown in Additional file 2.

### Across breed general trends between 5-year blocks

**Fig. 4:**
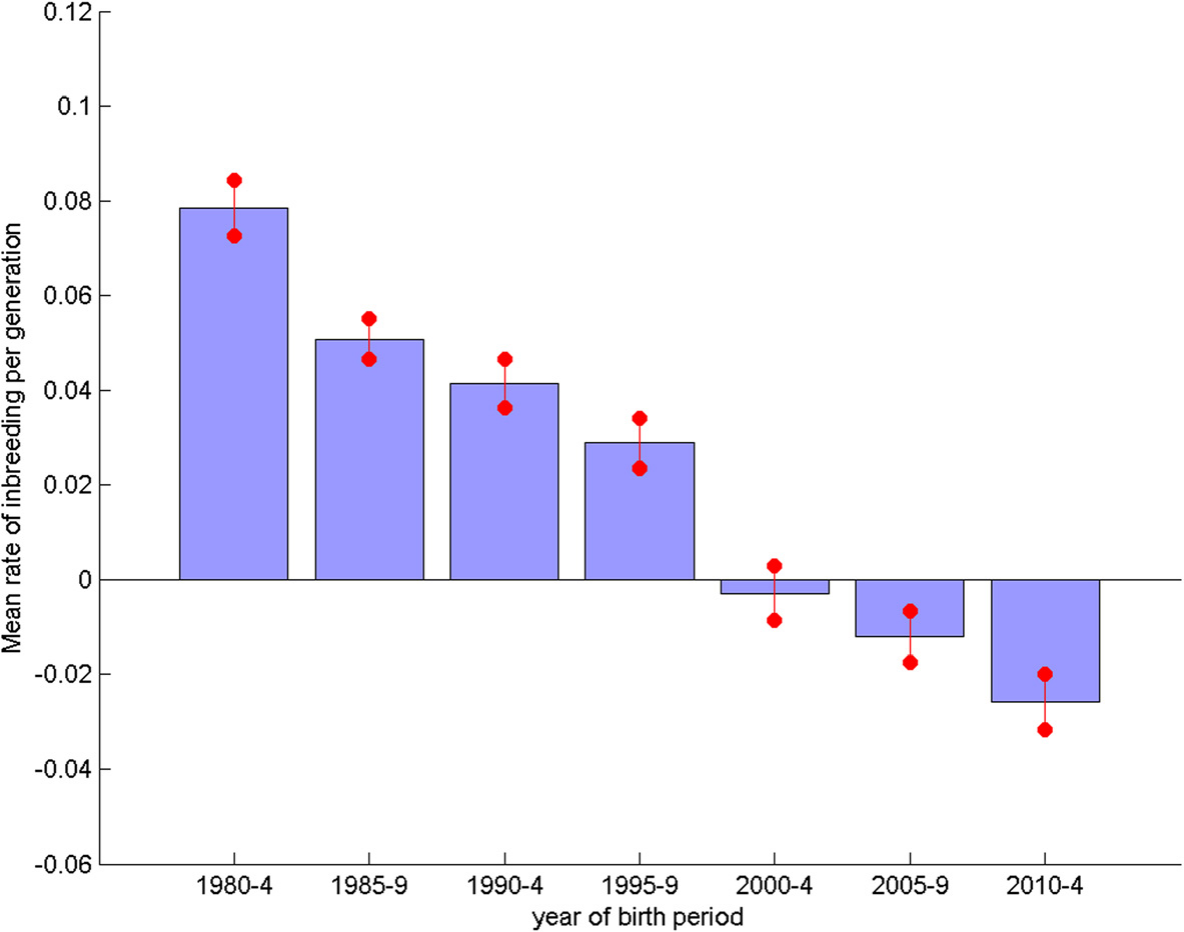
Mean rate of inbreeding per 5 year block. Mean rate of inbreeding per generation (ΔF) for breeds with an average of >50 registrations per year per 5 year block (*n* = 121), for each of the 5 year periods from 1980–2014. Error bars indicate the standard error of the mean

Where there are sufficient data a table displaying sire and dam usage statistics and genetic parameters over each of the seven 5-year blocks is included in breed reports. The mean rate of inbreeding (per generation, ΔF) over all breeds with an average of >50 registrations per annum in each of the seven 5-year blocks differed significantly over the blocks (*P* < 0.001, ANOVA). Fig. 4 shows a clear declining trend in across breed mean ΔF over successive 5-year blocks, revealing that the greatest decline in genetic diversity (when ΔF is largest) across breeds occurred in the 1980s and 1990s. Since 2000 the general trend in ΔF has been negative, implying an apparent increase in genetic diversity.

**Fig. 5:**
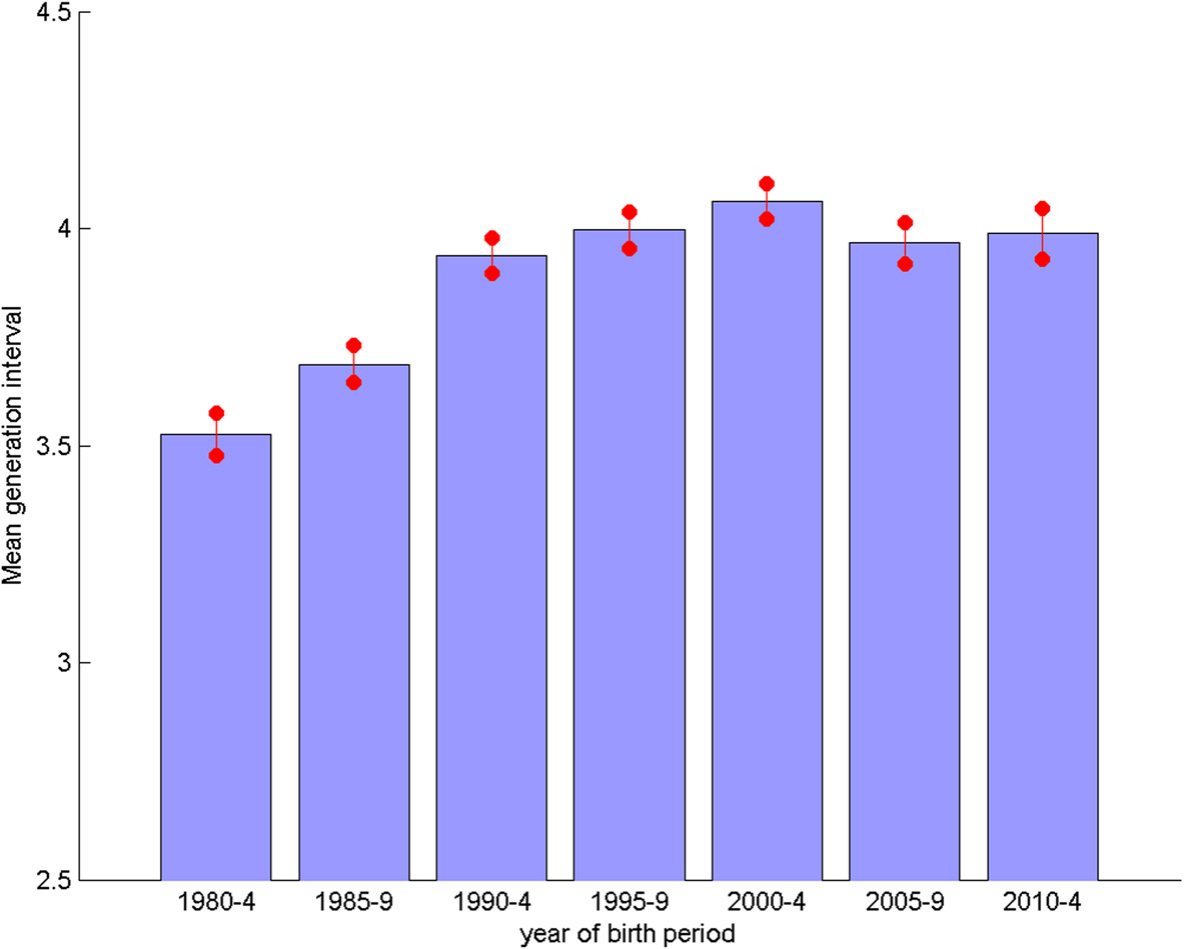
Mean generation interval per 5 year block. Mean generation interval (*L*) for breeds with an average of >50 registrations per year per 5 year block (*n* = 121), for each of the 5 year blocks from 1980–2014. Error bars indicate the standard error of the mean

A general increase in the generation interval (*L*) from the 1980s to the 1990s onwards was observed (Fig. 5, *P* < 0.001, ANOVA). This may partially account for the declining general trend in mean ΔF in the periods 1980–1984 and 1985–1989, where the difference in *L* is greatest. Mean *L* is not significantly different over blocks 1990–1994 to 2010–2014 (*P* = 0.42, ANOVA). This observation implies that the continued declining trend in mean ΔF is not solely due to changes in *L*, and so is in part due to additional changes in breeding practice.

#### Contrast between numerically small and large breeds

**Fig. 6:**
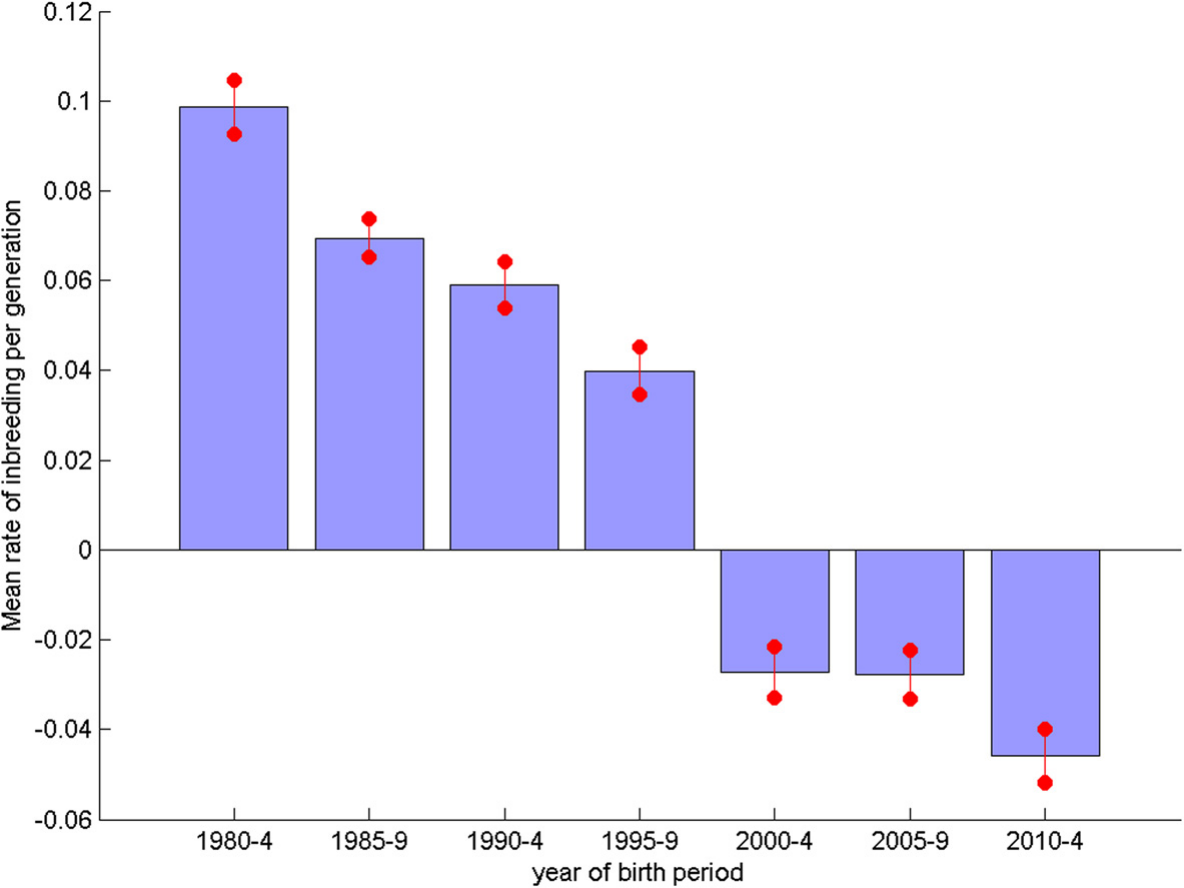
Mean rate of inbreeding per 5 year block of vulnerable native breeds. Mean rate of inbreeding per generation for vulnerable native breeds, for each of the 5 year periods from 1980–2014. Error bars indicate the standard error of the mean

**Fig. 7:**
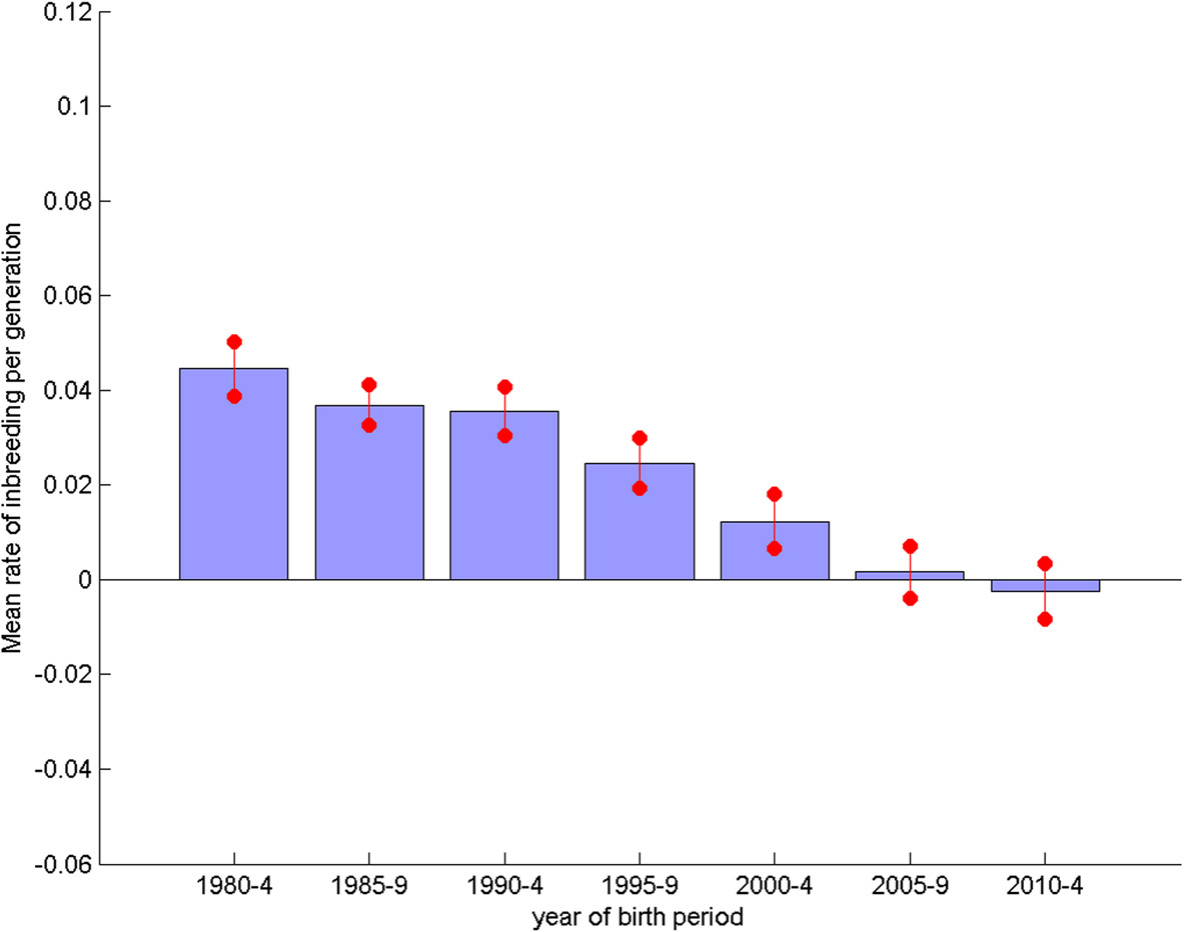
Mean rate of inbreeding per 5 year block of common breeds. Mean rate of inbreeding per generation for the 20 most common breeds 1980–2014, for each of the 5 year periods from 1980–2014. Error bars indicate the standard error of the mean

Comparison of the mean rate of inbreeding (per generation, ΔF) for 28 vulnerable native breeds (VNBs, ≤300 registrations in 2014, http://www.thekennelclub.org.uk/getting-a-dog-or-puppy/finding-the-right-dog/vulnerable-native-breeds/) and the 20 most common breeds (listed in Additional file 3) over the period 1980–2014 (Figs. 6 and 7 respectively) show the same trend of generally declining mean ΔF over the seven 5-year blocks. However, the magnitude of the change in mean ΔF is greater for the VNBs, with higher mean ΔF in block 1980–1984 and lower in 2010–2014 than the across breed average (Fig. 4), with the reverse occurring for the 20 most common breeds.

## Discussion

It is hoped that the public availability of population analysis reports for all 215 breeds currently recognised by the Kennel Club will enable breeders and other stakeholders to achieve a better understanding of the unique situation facing each breed. Both the descriptors of population dynamics (i.e. number of registrations, number of sires used etc.) and the genetic parameters (i.e. ΔF and N_e_) inform of the recent breed history and of the necessary considerations framing potential breeding strategies in the future. Different breeds with varying dynamics and levels of genetic diversity will have different options available in balancing meeting selection objectives and maintaining a sustainable ΔF. The breed reports are intended to provide a backdrop to discussions over the best approaches.

The plot of observed inbreeding for Labrador Retrievers (Fig. 2) is fairly typical of the profile of most breeds, with a steep incline in the 1980s gradually flattening to present, and in some breeds even recently declining. This corresponds to the general trend detected across all breeds (with sufficient numbers registered) of a high ΔF in the 1980s and 1990s, followed by a general decline to a negative ΔF latterly (Fig. 4, where the magnitude of the bars relates to the gradient of ‘observed inbreeding’ in Fig. 2). The across breeds mean ΔF in the blocks 1980–1984, 1985–1989, 1990–1994 and 1995–1999 all far exceed the recommended maximum of 0.01 per generation, above which the detrimental consequences of inbreeding are expected to be observed (i.e. N_e_ < 50; [11]). Thus, general breeding practices in the 1980s and 1990s appear to have resulted in a major contraction of within breed genetic diversity, while practices latterly have, to some extent, ameliorated this. The UK quarantine laws requiring immigrant dogs spend a six month period at quarantine kennels were relaxed in 2000 with the introduction of the Pet Passport scheme. It may be expected that the number of dogs imported to the UK after this date would have increased, and the use of apparently more distantly related migrant animals for breeding would contribute to the recent decline in ΔF. Although broadly the same declining trend in ΔF was observed in numerically small and large breeds (Fig. 6 and 7 respectively), the extent of the change was greater in the VNBs. It may be that the effects of general breeding practices were exacerbated in numerically small breeds (VNBs) in earlier years and that the increased availability of breeding stock from outside the UK from 2000 onwards provided a much needed injection of genetic diversity. In the case of the consistently more common breeds, the smaller mean ΔF in early years may reflect a larger pool of potential breeding animals, and the slower decline in mean ΔF due to a smaller proportional impact of migrants.

The whole period N_e_ varied widely across breeds, ranging from 23.8 to 918.8 (where determinable, in breeds with an average of >50 registrations per annum over each 5-year block). Individual breed whole period N_e_ represents the slope of ‘best fit’ through the ‘observed inbreeding’ plot (example in Fig. 2) over 1980–2014, describing the idealised population size that would be expected to exhibit the ΔF observed over 35 years. As such, it does not account for fluctuations in ΔF within that 35 year period, which have been shown in the results and discussed above. It is therefore important to take into account the individual breed profile in ‘observed inbreeding’ and ΔF and N_e_ over the 5-year blocks to determine the extent of contraction in genetic diversity, when it occurred, and the degree to which this may have been restored more recently to inform future breeding strategies on a breed-by-breed basis.

The whole period N_e_ was independent of census population size (as judged by mean annual registrations); some very numerous breeds had a small whole period N_e_ (e.g. English Springer Spaniel, mean annual registrations = 10,885.7, N_e_ = 45) while some much rarer breeds had a relatively high whole period N_e_ (e.g. Sealyham Terrier, mean annual registrations = 87.1, N_e_ = 111). A possible reason for low N_e_ in numerically large breeds is underlying population ‘sub-structure’. In common breeds, such as the English Springer Spaniel, several ‘sub-populations’ are likely to exist; for example working, show and pet populations, and even geographically localised populations. The existence of sub-populations (to whatever degree of independence) is indeterminable in this analysis of pedigree data. Breeding practices within each of multiple independent sub-populations giving rise to a positive ΔF in each will lead to a positive breed-wide ΔF. However, this breed-wide figure represents [mean] loss of genetic diversity within each sub-population, but ignores the fact that there is actually an increase in genetic diversity between sub-populations due to drift acting on allele frequencies [9]. Thus the breed-wide estimate of ΔF and N_e_ fails to take account of the between sub-population genetic variation, which is easily tapped by migration between sub-populations. Therefore, for some of the numerically larger breeds with show and working ‘types’ , the breed-wide whole period N_e_ may belie the amount of true within breed genetic diversity.

Although the lack of complete pedigree data beyond a certain number of generations for migrant animals hampers the ability to fully determine co-ancestry where it does exist (therefore potentially underestimating ΔF and N_e_ for breeds making wide use of imported animals), the principle outlined above also applies to breed populations in different countries. Even where such migrant animals originally trace back to the UK (for example the English Setter), populations which have existed in semi-isolation in different countries will be subject to the effects of drift, potentially increasing genetic diversity between sub-populations while it simultaneously declines within each. However, to what degree the increase in between sub-population diversity counteracts the potential underestimation of ΔF and N_e_ due to incomplete pedigree information is unknown.

The sustainable ΔF and N_e_ observed in some numerically smaller breeds (e.g. Dandie Dinmont Terrier, Cardigan Welsh Corgi, Sealyham Terrier, Bloodhound) would appear to be related to the effective management of genetic diversity. The plots of observed and expected inbreeding in these breeds show small divergence between the two, implying only a slight departure from random mating (Additional file 4: Figure S1). This may be due to heightened breeder awareness of the importance of conserving genetic diversity in numerically small populations. However, in some cases where the breed is numerically small, effective management of genetic diversity may not be enough. Otterhound breeders appear to have been managing genetic diversity as effectively as possible over 1980–2014 (judged by the conformity of the plots of observed and expected inbreeding), and so the high ΔF and low N_e_ observed may be due to small actual population size (Additional file 5: Figure S2). There are methods which achieve lower ΔF than predicted via random mating which may be useful in the preservation of genetic diversity, for example negative assortative mating and optimum selection techniques where weighting is only placed on minimising ΔF [12, 13]. However, these methods rely on tightly co-ordinated decisions being made on breeding animals, which is unlikely even in the rarest of breeds.

Furthermore, preserving genetic diversity (via a sustainable ΔF) is a single, albeit important, objective among many in the promotion of health and welfare in dog breeds. Many breeds face significant welfare problems due to a high burden of inherited disease [4] and extreme conformation [14], for which a lasting and widespread improvement can only be achieved via selection. Selection requires genetic variation to be present within the population meaning that the desired response will be more difficult to achieve in populations with a high ΔF. Partial restoration of genetic variation will occur via mutation, but the rate is of mutation is small, the genomic location random and there is strong selection pressure against mutations in coding regions of the genome, where mutations lead to malfunction or loss of function. Therefore, where genetic variation is depleted in a population, migration of breeding animals from a different population is the only practical means of regeneration to enable selection. Selection and inbreeding have been demonstrated to be fundamentally related via genetic contributions [6–8], meaning that balancing the competing objectives of genetic gain and sustainable ΔF is vital to the welfare and sustainability of many breeds. The intrinsic relationship between selection and inbreeding means that in numerically larger breeds the ΔF observed in these analyses could be considered to be a ‘signature’ of selection, although the objectives and traits under selection remain unknown. Therefore, breeds with very low N_e_ despite moderate to high mean registrations per year (e.g. Airedale Terrier, Bearded Collie, Irish Setter, Yorkshire Terrier; Additional file 6: Figure S3) might be considered to have been subject to relatively intense selection over the past 35 years.

While the use of inbreeding coefficients to derive ΔF in a breed or population provides a useful indication of past practice, they remain a retrospective measure of co-ancestry. Well-intentioned but widespread use of unrelated animals in breeding programmes in an attempt to increase genetic diversity, while reducing mean F in the next generation, can lead to such individuals becoming popular sires. Popular sires make a large genetic contribution to future generations of the breed, and are therefore the major contributor to a high ΔF over subsequent generations [15]. Breeders of pedigree breeds making particular use of migrant animals must be aware of this. Using a greater proportion of males for breeding will help mitigate the effect popular sires have on ΔF, but the common practice of substituting a known popular sire with a close male relative in a potential mating will have limited impact on minimising rises in future ΔF, due to shared genetics. The monitoring of genetic contributions may allow prospective identification of potential over-popular sires and relatives, and research into how such a strategy may be tailored to dog breeding is ongoing [16].

## Conclusions

The general trend detected across breeds was that ΔF was highest in the 1980s and 1990s, implying a high rate of loss of genetic diversity within most breeds. However, since 2000 ΔF tends to have decreased, even becoming negative in some cases, indicating a slowing of the rate of loss or even some moderate restoration of genetic variation. This change in trend may have been influenced by the increased availability of migrants since changes to UK quarantine laws in 2000.

Most breeds show extensive use of popular sires. The theory of genetic contributions, which demonstrates the fundamental relationship of inbreeding and selection, implies that popular sires are the major contributor to high ΔF. Breeders should guard against the over-use of sires as a strategy to maintain sustainable ΔF in the breed.

## Methods

All electronically recorded pedigree data held by the Kennel Club was used to determine population statistics per year from 1980–2014 for each of the 215 recognised breeds (data extracted 9^th^ February 2015).

### Number of registrations

The number of registered animals born per year was recorded, and (where sufficient numbers justified) regressed on year of birth, the regression coefficient describing the overall trend in breed registrations over the 35 year period.

### Variation in sire usage

Over each year 1980–2014 the total number of unique sires used was determined, and the maximum, mean, median, mode and standard deviation in number of progeny per sire computed. The percentage of registered animals born per year to the most prolific 50 %, 25 %, 10 % and 5 % of sires was calculated to describe the extent of ‘popular sire’ usage in each breed.

### Generation interval

The generation interval is the mean age of parents at the birth of progeny which themselves go on to reproduce [17]. The mean age in days of sires and of dams at the birth of breeding progeny born in each year 1980–2014 was calculated. The overall breed mean generation interval (*L*) was computed as the mean of generation intervals calculated per year from 1980–2014, and is quoted in years.

### Genetic diversity

Inbreeding coefficients for all animals in each breed pedigree were calculated using the algorithm of Meuwissen and Luo [18]. Mean inbreeding coefficients for all animals born in each year were computed, and plotted against year of birth. The rate of inbreeding per annum was calculated as the regression coefficient of ln(1-F_t_) on year of birth, where F_t_ is the mean inbreeding coefficient of animals born in year *t*. The rate of inbreeding per annum was multiplied by the breed mean generation interval (*L*) to obtain the rate of inbreeding per generation (ΔF). The effective population size (N_e_) was calculated for each breed over the period 1980–2014 as (2 ΔF)^−1^ [5].

The coefficient of kinship between two animals is equal to the inbreeding coefficient of offspring of those two animals [19]. Thus, mean kinship coefficient of all or a random sample of animals yields an estimate of mean inbreeding coefficient from random mating. Annual estimates of mean kinship coefficient (1980–2014) were termed ‘expected inbreeding’ and plotted alongside ‘observed inbreeding’, staggered by breed generation interval (*L*, since animals born in year *t* would on average produce breeding progeny at *t + L* in the future). Calculation of coefficient of kinship per year of birth is computationally intensive for large populations, since the number of calculations is *½(n*^*2*^*-n)* (where *n* is the number of animals in the sample) and so the method used was dependent on the number of animals born in a particular year. Where ≤500 animals were born in a particular year, all kinships were calculated and the mean computed. Where >500 and ≤2000 animals were born in a year, kinships of a single, random sample of 500 animals were calculated and the mean computed. Where >2000 animals were born in a year, kinships were calculated for 10 samples of 50 randomly selected animals. The overall mean and standard deviation of the 10 sample means were calculated. In this final scenario, the standard deviation of samples mean kinships is indicated by the ‘bleeding’ of the line denoting expected inbreeding.

### Differences between 5-year blocks within the period 1980–2014

The statistics described above were also calculated over seven 5-year blocks (1980–1984, 1985–1989, 1990–1994, 1995–1999, 2000–2004, 2005–2009, 2010–2014) to provide more detail of changing trends within the overall 35 year period. Where the numerical size of the breed sufficed (mean of >50 registrations per annum in each of the seven 5-year blocks), differences in across breeds mean ΔF and *L* between the 5-year blocks were tested for statistical significance using ANOVA, which would indicate a change in general (across breed) trends.

## Additional files

Additional file 1:
**Whole period (1980–2014) rate of inbreeding per annum (multiply by 100 for percentage), mean generation interval (** ***L*** **) and effective population size (N**_**e**_ **) for the 121 breeds with an average of >50 registrations in each of the seven** **5-year** **blocks 1980–1984, 1985–1989, 1990–1994, 1995–1999, 2000–2004, 2005–2009, 2010–2014.** ‘n/a’ indicates a declining rate of inbreeding (increasing genetic diversity), meaning effective population size is incalculable. (DOCX 22 kb) Additional file 2:
**Whole period (1980–2014) rate of inbreeding per annum (dF, multiply by 100 for percentage), mean generation interval (L), effective population size (N**_**e**_ **) and mean registrations over the seven** **5-year** **blocks (1980–1984, 1985–1989, 1990–1994, 1995–1999, 2000–2004, 2005–2009, 2010–2014) for the 94 breeds with an average of <50 registrations in at least one of those blocks.** ‘n/a’ in columns dF, L and N_e_ indicates too few data to yield meaningful results. ‘n/a’ in the N_e_ column only indicates a negative rate of inbreeding over the whole period, meaning N_e_ is indeterminable. (DOCX 32 kb) Additional file 3:
**List of Vulnerable Native Breeds (VNBs) as of 2014 and the 20 most common breeds over the period 1980–2014.** (DOCX 19 kb) Additional file 4: Figure S1. The plots of observed and expected inbreeding for four breeds showingsmall divergence between the two. (PNG 204 kb) Additional file 5: Figure S2. Plot of observed and expected inbreeding for the Otterhound breed, showingsmall divergence but a rise in both. (PNG 25 kb) Additional file 6: Figure S3. The plots of observed and expected inbreeding for four breeds showing a steeprise in observed inbreeding, and therefore a low effective population size (Ne). (PNG 183 kb)
