# Supplementary material for: Trends in genetic diversity for all Kennel Club registered pedigree dog breeds
Source: Canine Genet Epidemiol. 2015 Sep 21;2:13. doi: 10.1186/s40575-015-0027-4 (PMC4579366; doi:10.1186/s40575-015-0027-4)
Supplement: Additional file 1: — Whole period (1980–2014) rate of inbreeding per annum (multiply by 100 for percentage), mean generation interval ( L ) and effective population size (N e ) for the 121 breeds with an average of >50 registrations in each of the seven 5-year blocks 1980–1984, 1985–1989, 1990–1994, 1995–1999, 2000–2004, 2005–2009, 2010–2014. ‘n/a’ indicates a declining rate of inbreeding (increasing genetic diversity), meaning effective population size is incalculable. (DOCX 22 kb) [file 40575_2015_27_MOESM1_ESM.docx]

**Appendix 1: Whole period (1980-2014) rate of inbreeding per annum (multiply by 100 for percentage), mean generation interval (*L*) and effective population size (**N_e_**) for the 121 breeds with an average of >50 registrations in each of the seven 5-year blocks 1980-4, 1985-9, 1990-4, 1995-9, 2000-4, 2005-9, 2010-4. ‘n/a’ indicates a declining rate of inbreeding (increasing genetic diversity), meaning effective population size is incalculable.**

| Breed | Rate of inbreeding per annum | Generation interval (years) | Effective population size (N_e_) |
| --- | --- | --- | --- |
| Afghan Hound | 0.00041 | 5.01 | 245.05 |
| Airedale Terrier | 0.00486 | 4.02 | 25.61 |
| Basset Griffon Vendeen Petit | 0.00240 | 3.69 | 56.58 |
| Basset Hound | 0.00181 | 3.72 | 74.15 |
| Beagle | 0.00236 | 3.86 | 54.93 |
| Bearded Collie | 0.00415 | 5.04 | 23.91 |
| Bedlington Terrier | 0.00246 | 4.21 | 48.32 |
| Belgian Shepherd Dog (Groendendael) | 0.00026 | 3.97 | 480.56 |
| Belgian Shepherd Dog (Tervueren) | 0.00050 | 4.11 | 243.42 |
| Bernese Mountain Dog | -0.00024 | 3.62 | n/a |
| Bichon Frise | 0.00181 | 3.43 | 80.24 |
| Bloodhound | 0.00014 | 3.88 | 890.49 |
| Border Collie | 0.00106 | 4.14 | 113.54 |
| Border Terrier | 0.00127 | 3.71 | 106.08 |
| Borzoi | 0.00013 | 4.35 | 918.83 |
| Boston Terrier | 0.00411 | 3.31 | 36.78 |
| Bouvier Des Flandres | 0.00099 | 3.80 | 133.13 |
| Boxer | 0.00169 | 3.67 | 80.93 |
| Briard | -0.00035 | 4.32 | n/a |
| Bull Terrier | 0.00385 | 3.11 | 41.86 |
| Bull Terrier (Miniature) | 0.00136 | 3.06 | 119.89 |
| Bulldog | 0.00233 | 3.16 | 67.85 |
| Bullmastiff | 0.00151 | 3.25 | 101.47 |
| Cairn Terrier | 0.00181 | 3.93 | 70.30 |
| Cavalier King Charles Spaniel | 0.00129 | 3.50 | 111.16 |
| Chihuahua (Long Coat) | 0.00081 | 3.52 | 175.96 |
| Chihuahua (Smooth Coat) | 0.00187 | 3.30 | 81.05 |
| Chinese Crested | 0.00097 | 3.24 | 159.31 |
| Chow Chow | 0.00100 | 3.77 | 132.59 |
| Collie (Rough) | 0.00334 | 3.80 | 39.43 |
| Collie (Smooth) | 0.00136 | 4.10 | 90.03 |
| Dachshund (Long-Haired) | 0.00314 | 4.03 | 39.48 |
| Dachshund (Miniature Long-Haired) | 0.00155 | 3.61 | 89.16 |
| Dachshund (Miniature Wire-Haired) | 0.00130 | 3.49 | 110.39 |
| Dachshund (Miniature Smooth-Haired) | 0.00151 | 3.39 | 97.65 |
| Dachshund (Smooth-Haired) | 0.00210 | 4.03 | 59.07 |
| Dachshund (Wire-Haired) | 0.00043 | 3.87 | 298.23 |
| Dalmatian | 0.00085 | 4.14 | 142.41 |
| Dandie Dinmont Terrier | 0.00059 | 4.34 | 196.57 |
| Deerhound | 0.00212 | 3.94 | 60.04 |
| Dobermann | 0.00102 | 3.69 | 133.40 |
| English Setter | 0.00389 | 4.32 | 29.79 |
| Fox Terrier (Smooth) | 0.00157 | 3.97 | 80.40 |
| Fox Terrier (Wire) | 0.00329 | 3.61 | 42.07 |
| French Bulldog | 0.00118 | 3.20 | 132.28 |
| German Shepherd Dog | 0.00090 | 3.79 | 147.51 |
| German Shorthaired Pointer | 0.00127 | 4.61 | 85.72 |
| German Wirehaired Pointer | 0.00029 | 4.20 | 416.94 |
| Giant Schnauzer | 0.00134 | 3.73 | 100.02 |
| Gordon Setter | 0.00089 | 4.50 | 125.15 |
| Great Dane | 0.00090 | 3.34 | 166.77 |
| Griffon Bruxellois | 0.00215 | 3.50 | 66.48 |
| Hungarian Vizsla | 0.00062 | 4.66 | 173.92 |
| Irish Red And White Setter | 0.00319 | 3.98 | 39.41 |
| Irish Setter | 0.00401 | 4.57 | 27.26 |
| Irish Terrier | 0.00109 | 3.97 | 115.06 |
| Irish Wolfhound | 0.00063 | 3.59 | 222.22 |
| Italian Greyhound | 0.00145 | 4.25 | 80.92 |
| Japanese Chin | 0.00173 | 3.56 | 81.39 |
| Japanese Spitz | 0.00248 | 3.48 | 57.87 |
| Keeshond | 0.00025 | 4.00 | 491.94 |
| Kerry Blue Terrier | 0.00326 | 3.61 | 42.60 |
| King Charles Spaniel | 0.00338 | 3.55 | 41.64 |
| Lakeland Terrier | 0.00356 | 3.71 | 37.82 |
| Lancashire Heeler | 0.00391 | 3.90 | 32.78 |
| Large Munsterlander | 0.00188 | 4.44 | 59.77 |
| Lhaso Apso | 0.00282 | 3.47 | 51.15 |
| Lowchen Little Lion Dog | 0.00420 | 3.70 | 32.16 |
| Maltese | 0.00102 | 3.61 | 135.87 |
| Manchester Terrier | 0.00483 | 4.35 | 23.83 |
| Mastiff | 0.00211 | 3.36 | 70.55 |
| Miniature Pinscher | 0.00259 | 3.57 | 54.01 |
| Miniature Schnauzer | 0.00088 | 3.57 | 158.95 |
| Newfoundland | 0.00047 | 3.83 | 276.27 |
| Norfolk Terrier | 0.00301 | 3.59 | 46.20 |
| Norwegian Elkhound | 0.00055 | 4.01 | 225.55 |
| Norwich Terrier | 0.00301 | 3.59 | 46.20 |
| Old English Sheepdog | 0.00220 | 4.44 | 51.03 |
| Papillon | 0.00109 | 3.71 | 123.61 |
| Pekingese | 0.00239 | 3.43 | 61.01 |
| Pointer | 0.00195 | 4.16 | 61.53 |
| Pomeranian | 0.00140 | 3.62 | 99.07 |
| Poodle (Miniature) | 0.00098 | 3.87 | 131.44 |
| Poodle (Standard) | -0.00006 | 3.88 | n/a |
| Poodle (Toy) | 0.00099 | 3.69 | 136.31 |
| Pug | 0.00119 | 3.14 | 133.88 |
| Pyrenean Mountain Dog | 0.00147 | 4.12 | 82.54 |
| Retriever (Curly Coated) | 0.00361 | 4.24 | 32.68 |
| Retriever (Flat Coated) | 0.00161 | 4.58 | 67.91 |
| Retriever (Golden) | 0.00195 | 4.19 | 61.32 |
| Retriever (Labrador) | 0.00139 | 4.39 | 81.75 |
| Rhodesian Ridgeback | -0.00006 | 3.99 | n/a |
| Rottweiler | 0.00069 | 3.78 | 190.82 |
| Saluki | 0.00051 | 4.95 | 199.22 |
| Samoyed | 0.00190 | 4.06 | 64.72 |
| Schnauzer | 0.00121 | 3.92 | 105.42 |
| Scottish Terrier | 0.00104 | 3.82 | 125.94 |
| Sealyham Terrier | 0.00110 | 4.10 | 111.13 |
| Shetland Sheepdog | 0.00158 | 4.08 | 77.61 |
| Shih Tzu | 0.00081 | 3.63 | 170.09 |
| Siberian Husky | 0.00089 | 3.94 | 142.93 |
| Soft Coated Wheaten Terrier | 0.00058 | 4.47 | 193.68 |
| Spaniel (American Cocker) | 0.00047 | 3.53 | 300.25 |
| Spaniel (Clumber) | 0.00530 | 3.85 | 24.49 |
| Spaniel (Cocker) | 0.00265 | 3.84 | 49.10 |
| Spaniel (English Springer) | 0.00249 | 4.45 | 45.08 |
| Spaniel (Field) | 0.00379 | 4.12 | 31.96 |
| Spaniel (Irish Water) | 0.00167 | 4.22 | 71.02 |
| Spaniel (Sussex) | 0.00380 | 4.08 | 32.24 |
| Spaniel (Welsh Springer) | 0.00315 | 4.35 | 36.45 |
| St Bernard | 0.00116 | 3.31 | 130.62 |
| Staffordshire Bull Terrier | 0.00155 | 3.30 | 97.71 |
| Tibetan Spaniel | 0.00271 | 4.22 | 43.67 |
| Tibetan Terrier | -0.00221 | 4.10 | n/a |
| Weimaraner | 0.00165 | 4.01 | 75.40 |
| Welsh Corgi (Cardigan) | 0.00093 | 4.27 | 125.57 |
| Welsh Corgi (Pembroke) | 0.00239 | 3.71 | 56.42 |
| Welsh Terrier | 0.00398 | 3.55 | 35.36 |
| West Highland Terrier | 0.00144 | 3.85 | 90.47 |
| Whippet | 0.00213 | 4.15 | 56.43 |
| Yorkshire Terrier | 0.00306 | 4.06 | 40.24 |
