# Supplementary material for: Trends in genetic diversity for all Kennel Club registered pedigree dog breeds
Source: Canine Genet Epidemiol. 2015 Sep 21;2:13. doi: 10.1186/s40575-015-0027-4 (PMC4579366; doi:10.1186/s40575-015-0027-4)
Supplement: Additional file 2: — Whole period (1980–2014) rate of inbreeding per annum (dF, multiply by 100 for percentage), mean generation interval (L), effective population size (N e ) and mean registrations over the seven 5-year blocks (1980–1984, 1985–1989, 1990–1994, 1995–1999, 2000–2004, 2005–2009, 2010–2014) for the 94 breeds with an average of <50 registrations in at least one of those blocks. ‘n/a’ in columns dF, L and Ne indicates too few data to yield meaningful results. ‘n/a’ in the Ne column only indicates a negative rate of inbreeding over the whole period, meaning Ne is indeterminable. (DOCX 32 kb) [file 40575_2015_27_MOESM2_ESM.docx]

**Appendix 2: Whole period (1980-2014) rate of inbreeding per annum (dF, multiply by 100 for percentage), mean generation interval (L), effective population size (**N_e_**) and mean registrations over the seven 5-year blocks (1980-4, 1985-9, 1990-4, 1995-9, 2000-4, 2005-9, 2010-4) for the 94 breeds with an average of <50 registrations in at least one of those blocks. ‘n/a’ in columns dF, L and** N_e_ **indicates too few data to yield meaningful results. ‘n/a’ in the** N_e_ **column only indicates a negative rate of inbreeding over the whole period, meaning** N_e_ **is indeterminable.**

| Breed | dF | L | N_e_ | Mean registrations per 5-year block | | | | | | |
| --- | --- | --- | --- | --- | --- | --- | --- | --- | --- | --- |
|  |  |  |  | 1980-4 | 1985-9 | 1990-1 | 1995-9 | 2000-4 | 2005-9 | 2010-4 |
| Affenpinscher | 0.00090 | 3.60 | 153.56 | 19 | 40 | 81.4 | 105.4 | 143.4 | 135 | 95 |
| Akita | 0.00315 | 2.97 | 53.34 | 31 | 450.6 | 636 | 1325.4 | 1467.8 | 1231.4 | 741 |
| Alaskan Malamute | -0.00036 | 3.42 | n/a | 15 | 34.6 | 51.2 | 89.4 | 314 | 1076.4 | 1010.8 |
| Anatolian Shepherd Dog | 0.00080 | 4.14 | 150.62 | 38 | 108.6 | 96.6 | 51 | 49.6 | 47.8 | 22.2 |
| Australian Cattle Dog | 0.00099 | 3.76 | 134.00 | 13.6 | 17.8 | 18.2 | 42 | 65.6 | 85.6 | 57.2 |
| Australian Shepherd | -0.00082 | 3.93 | n/a | 19.4 | 23.2 | 45 | 69.6 | 111.4 | 163.6 | 166.4 |
| Australian Silky Terrier | 0.00002 | 3.88 | 5760.08 | 33 | 44 | 31.2 | 28.2 | 22 | 28.2 | 16.4 |
| Australian Terrier | 0.00144 | 3.88 | 89.88 | 68.4 | 56.8 | 49 | 51.4 | 45.4 | 59 | 34.2 |
| Azawakh | n/a | n/a | n/a | 0 | 0 | 0 | 0.4 | 2.4 | 2.8 | 1 |
| Basenji | -0.00047 | 4.57 | n/a | 85.4 | 92 | 77.4 | 64.2 | 63.2 | 53.6 | 44.6 |
| Basset Bleu De Gasgogne | n/a | n/a | n/a | 0 | 0 | 0.4 | 4.6 | 5 | 3.4 | 3 |
| Basset Fauve De Bretagne | 0.00133 | 3.71 | 101.13 | 1.4 | 4.8 | 20 | 37.6 | 69.8 | 96.4 | 99.2 |
| Basset Griffon Vendeen Grand | 0.00154 | 3.34 | 97.47 | 0 | 1 | 9.8 | 62.2 | 118.4 | 94.2 | 105.4 |
| Bavarian Mountain Dog | n/a | n/a | n/a | 0 | 0.2 | 2.2 | 5.4 | 2.4 | 26.6 | 50.8 |
| Beauceron | n/a | n/a | n/a | 0 | 0 | 0.4 | 9.2 | 11.2 | 18.6 | 17 |
| Belgian Shepherd Dog (Laekenois) | n/a | n/a | n/a | 2.2 | 7 | 7.2 | 0.4 | 3.4 | 7 | 5 |
| Belgian Shepherd Dog (Malinois) | -0.00012 | 3.92 | n/a | 18.4 | 11.4 | 21.8 | 25.4 | 57.2 | 97.6 | 133.6 |
| Bergomasco | n/a | n/a | n/a | 0 | 0.6 | 2.6 | 2.6 | 4 | 1.6 | 5.2 |
| Bolognese | -0.00568 | 3.22 | n/a | 0 | 2.4 | 23.8 | 36.8 | 50.2 | 107 | 137.8 |
| Bracco Italiano | -0.00413 | 2.88 | n/a | 0 | 0.8 | 4.4 | 10.6 | 19.6 | 49.6 | 71.8 |
| Brittany | 0.00135 | 3.55 | 104.53 | 17.8 | 71 | 113.8 | 133.4 | 116 | 151.4 | 125.2 |
| Canaan Dog | n/a | n/a | n/a | 0.2 | 5.8 | 6.6 | 8 | 18.8 | 14.8 | 12.2 |
| Canadian Eskimo Dog | n/a | n/a | n/a | 0 | 0 | 0 | 0.8 | 4.4 | 7 | 9.2 |
| Catalan Sheepdog | n/a | n/a | n/a | 0 | 0 | 0 | 0.4 | 1.8 | 19 | 37.4 |
| Cesky Terrier | 0.00885 | 3.30 | 17.11 | 0.2 | 1 | 10.2 | 34.2 | 34.6 | 42.8 | 26.6 |
| Cirneco Dell'etna | n/a | n/a | n/a | 0 | 0 | 0 | 0.4 | 2.2 | 10.6 | 14.6 |
| Coton De Tulear | -0.00125 | 2.53 | n/a | 0 | 0.6 | 0 | 3.4 | 21.6 | 102.2 | 220.4 |
| Dogue De Bordeaux | 0.00139 | 2.37 | 151.53 | 0 | 0 | 6.4 | 130.2 | 1000 | 2494.4 | 2473.6 |
| English Toy Terrier | 0.00095 | 4.01 | 131.57 | 43.2 | 53.6 | 54.4 | 52.4 | 73.6 | 120.2 | 112.2 |
| Entlebucher Mountain Dog | n/a | n/a | n/a | 0 | 0 | 0 | 0 | 1 | 2.2 | 9.6 |
| Estrela Mountain Dog | 0.00112 | 3.29 | 136.19 | 12 | 9.8 | 15 | 17.4 | 29.8 | 34.4 | 22.6 |
| Eurasier | -0.00081 | 2.70 | n/a | 0 | 0 | 0 | 0.4 | 11.8 | 80.4 | 134.8 |
| Finnish Lapphund | 0.00067 | 3.74 | 198.26 | 0 | 0.8 | 5.6 | 24.4 | 27.6 | 51.4 | 64.6 |
| Finnish Spitz | -0.00092 | 4.82 | n/a | 58 | 67.4 | 33.8 | 25.8 | 26.6 | 30.4 | 18.6 |
| Foxhound | n/a | n/a | n/a | 0.8 | 0.2 | 2.2 | 0.6 | 4.8 | 6.6 | 4.8 |
| German Longhaired Pointer | n/a | n/a | n/a | 0 | 0.2 | 1 | 9.4 | 17.6 | 24.4 | 19.4 |
| German Pinscher | n/a | n/a | n/a | 14 | 23 | 14 | 28.6 | 21.6 | 13.6 | 19.8 |
| German Spitz (Klein) | 0.00239 | 3.36 | 62.29 | 4.8 | 62.4 | 98.4 | 132.4 | 126.4 | 153.6 | 111.4 |
| German Spitz (Mittel) | 0.00349 | 3.22 | 44.44 | 1 | 33.6 | 84.6 | 103.6 | 90.8 | 82.4 | 70 |
| Glen Of Imaal Terrier | 0.00240 | 3.65 | 57.15 | 28.8 | 44.8 | 59.8 | 70.8 | 39.4 | 53.8 | 61.6 |
| Grand Bleu De Gascogne | n/a | n/a | n/a | 0.2 | 4.8 | 11.6 | 11 | 4.6 | 6.6 | 0.2 |
| Greater Swiss Mountain Dog | n/a | n/a | n/a | 0 | 0 | 0 | 0 | 0.6 | 8 | 30.2 |
| Greenland Dog | n/a | n/a | n/a | 3 | 3.2 | 3.6 | 4.6 | 8.4 | 9.6 | 6.4 |
| Greyhound | -0.00130 | 4.33 | n/a | 50.8 | 63.4 | 70.2 | 64 | 63.6 | 49.4 | 29.6 |
| Griffon Fauvre De Bretagne | n/a | n/a | n/a | 0 | 0 | 0 | 0 | 0 | 0.8 | 2.6 |
| Hamiltonstovare | n/a | n/a | n/a | 3.2 | 9.4 | 12.2 | 23.2 | 26.4 | 17.2 | 13.6 |
| Havanese | -0.00313 | 2.89 | n/a | 0 | 0 | 7.4 | 39.4 | 60.8 | 130.4 | 238 |
| Hovawart | -0.00086 | 4.18 | n/a | 6 | 13.2 | 11.4 | 27.2 | 26 | 39.2 | 23 |
| Hungarian Kuvasz | n/a | n/a | n/a | 0 | 0.4 | 13 | 6 | 1 | 0.8 | 0.2 |
| Hungarian Puli | 0.00090 | 4.75 | 117.27 | 60.2 | 78.4 | 88 | 132.8 | 74.4 | 69.8 | 47.4 |
| Hungarian Pumi | n/a | n/a | n/a | 0 | 0 | 0 | 0 | 0 | 0.2 | 5.6 |
| Hungarian Wirehaired Vizsla | 0.00057 | 3.23 | 270.72 | 0.2 | 0.2 | 6.8 | 49 | 124 | 286.2 | 479.8 |
| Ibizan Hound | n/a | n/a | n/a | 19 | 21.8 | 20.2 | 16.4 | 10.2 | 14.4 | 9.4 |
| Italian Spinone | 0.00107 | 3.36 | 139.51 | 15.2 | 119.6 | 231.6 | 368.4 | 334.6 | 425 | 462.4 |
| Japanese Akita | n/a | n/a | n/a | 0 | 0 | 0.2 | 1.2 | 6 | 32.4 | 71.2 |
| Japanese Shiba Inu | 0.00209 | 2.93 | 81.85 | 1.4 | 44 | 155.4 | 191 | 151 | 189.6 | 229.2 |
| Komondor | n/a | n/a | n/a | 2.8 | 9.2 | 7.4 | 10.4 | 8.8 | 8.8 | 7.2 |
| Kooikerhoundje | n/a | n/a | n/a | 0.2 | 3.6 | 9 | 12.2 | 7.6 | 13.4 | 25.2 |
| Korean Jindo | n/a | n/a | n/a | 0 | 0 | 0 | 0.2 | 2.6 | 7.4 | 13.2 |
| Korthals Griffon | n/a | n/a | n/a | 0 | 0 | 0 | 0 | 3.8 | 23.4 | 38.6 |
| Lagotto Romagnolo | n/a | n/a | n/a | 0 | 0 | 0 | 6.6 | 25.4 | 27.6 | 44.2 |
| Leonberger | 0.00019 | 3.48 | 759.44 | 4.8 | 20 | 60.4 | 224.4 | 315.2 | 388 | 359.2 |
| Maremma Sheepdog | -0.00042 | 3.85 | n/a | 51.2 | 54.2 | 45.4 | 39.6 | 26.4 | 23.4 | 28.4 |
| Mexican Hairless (intermediate) | n/a | n/a | n/a | 0 | 0.2 | 0 | 0.6 | 0.6 | 5.2 | 9.4 |
| Mexican Hairless (Miniature) | n/a | n/a | n/a | 0 | 0 | 0 | 0.2 | 0 | 0.6 | 8.2 |
| Mexican Hairless (Standard) | n/a | n/a | n/a | 0 | 0 | 0 | 1.8 | 1.2 | 0.6 | 5.2 |
| Neapolitan Mastiff | -0.00001 | 2.42 | n/a | 2.4 | 7.2 | 129.8 | 422.6 | 343.6 | 222.6 | 114.4 |
| Norwegian Buhund | 0.00228 | 4.20 | 52.11 | 88.4 | 76.6 | 38 | 38.6 | 38.6 | 22.8 | 26.4 |
| Otterhound | 0.00338 | 4.36 | 33.91 | 34.2 | 45.8 | 55.4 | 44.8 | 39.2 | 46.8 | 35.2 |
| Parson Russell Terrier | 0.00343 | 3.32 | 43.95 | 18.2 | 80.6 | 304.8 | 547 | 731.8 | 778 | 498.8 |
| Pharaoh Hound | -0.00013 | 4.93 | n/a | 27 | 29.4 | 27.6 | 27.6 | 25.4 | 28 | 28 |
| Picardy Sheepdog | n/a | n/a | n/a | 0 | 0 | 0 | 0 | 0.8 | 2 | 1.4 |
| Polish Lowland Sheepdog | 0.00316 | 3.76 | 42.16 | 0.6 | 28.8 | 100.6 | 76.8 | 54.6 | 62.6 | 55.8 |
| Portuguese Podengo | 0.00145 | 2.37 | 144.92 | 0 | 0 | 0 | 0.6 | 18.4 | 87 | 42.6 |
| Portuguese Pointer | n/a | n/a | n/a | 0 | 0 | 0 | 0 | 0.2 | 1.6 | 5.4 |
| Portuguese Water Dog | -0.00230 | 4.76 | n/a | 3.8 | 12.4 | 21.4 | 30.2 | 42.4 | 70 | 143.2 |
| Pyrenean Mastiff | n/a | n/a | n/a | 0 | 0 | 0 | 0.2 | 1.4 | 0.2 | 0 |
| Pyrenean Sheepdog | n/a | n/a | n/a | 0.4 | 1.8 | 7.6 | 16.4 | 25 | 22.6 | 18 |
| Retriever (Chesapeake Bay) | 0.00142 | 3.66 | 96.49 | 20 | 52.8 | 73.8 | 71.8 | 146.2 | 130.2 | 88 |
| Retriever (Nova Scotia Duck Tolling) | -0.00036 | 3.57 | n/a | 0 | 3.4 | 25 | 66.4 | 88 | 170.6 | 225 |
| Russian Black Terrier | 0.00393 | 3.18 | 40.06 | 0 | 0 | 0 | 10.8 | 82 | 94.8 | 65.8 |
| Schipperke | 0.00250 | 3.76 | 53.28 | 78 | 115.8 | 121.6 | 127.4 | 99.2 | 59.6 | 38.8 |
| Segugio Italiano | n/a | n/a | n/a | 1 | 2.2 | 4 | 3 | 2.6 | 1.2 | 0 |
| Shar Pei | -0.00123 | 2.81 | n/a | 36 | 173.4 | 484.4 | 857.6 | 1165.6 | 2064 | 1823.6 |
| Skye Terrier | 0.00116 | 4.48 | 96.11 | 106.2 | 106 | 111.6 | 88.8 | 70 | 55.6 | 43 |
| Sloughi | n/a | n/a | n/a | 9.8 | 1.8 | 7.2 | 12.2 | 15.4 | 9 | 9.2 |
| Slovakian Rough Haired Pointer | n/a | n/a | n/a | 0 | 0 | 0 | 2.2 | 9.8 | 53.6 | 49.4 |
| Small Munsterlander | n/a | n/a | n/a | 0 | 0 | 0 | 0.2 | 0.8 | 2.8 | 0.4 |
| Spaniel (American Water) | n/a | n/a | n/a | 0 | 0 | 0 | 0 | 0.2 | 0 | 0.4 |
| Spanish Water Dog | 0.00323 | 3.45 | 44.86 | 0 | 0.4 | 2.6 | 6.4 | 42.8 | 124.2 | 170 |
| Swedish Lapphund | n/a | n/a | n/a | 0 | 3.8 | 11 | 13.2 | 1.6 | 2.2 | 0.8 |
| Swedish Vallhund | -0.00118 | 4.47 | n/a | 39.2 | 63.2 | 74.2 | 55.8 | 41.8 | 45.2 | 41.8 |
| Tibetan Mastiff | -0.00146 | 3.76 | n/a | 3.2 | 21.6 | 17 | 22.6 | 34.4 | 54.4 | 65.8 |
| Turkish Kangal Dog | n/a | n/a | n/a | 0 | 0 | 0 | 0 | 1.4 | 2.6 | 8 |
