# Supplementary material for: Trends in genetic diversity for all Kennel Club registered pedigree dog breeds
Source: Canine Genet Epidemiol. 2015 Sep 21;2:13. doi: 10.1186/s40575-015-0027-4 (PMC4579366; doi:10.1186/s40575-015-0027-4)
Supplement: Additional file 3: — List of Vulnerable Native Breeds (VNBs) as of 2014 and the 20 most common breeds over the period 1980–2014. (DOCX 19 kb) [file 40575_2015_27_MOESM3_ESM.docx]

**Appendix 3: List of Vulnerable Native Breeds (VNBs) as of 2014 and the 20 most common breeds over the period 1980-2014.**

**Vulnerable Native Breeds (VNBs – alphabetical order):**

Bloodhound

Bull Terrier (Miniature)

Collie (Smooth)

Dandie Dinmont Terrier

Deerhound

English Toy Terrier

Fox Terrier (Smooth)

Glen of Imaal Terrier

Gordon Setter

Irish Red & White Setter

Irish Wolfhound

King Charles Spaniel

Kerry Blue Terrier

Lakeland Terrier

Lancashire Heeler

Manchester Terrier

Mastiff

Norwich Terrier

Otterhound

Retriever (Curly Coated)

Sealyham Terrier

Skye Terrier

Spaniel (Clumber)

Spaniel (Field)

Spaniel (Irish Water)

Spaniel (Sussex)

Welsh Corgi (Cardigan)

Welsh Corgi (Pembroke)

**Common breeds (ranked):**

1. Retriever (Labrador)
2. German Shepherd Dog
3. Spaniel (Cocker)
4. Spaniel (English Springer)
5. Retriever (Golden)
6. Cavalier King Charles Spaniel
7. West Highland Terrier
8. Yorkshire Terrier
9. Staffordshire Bull Terrier
10. Boxer
11. Rottweiler
12. Border Terrier
13. Shih Tzu
14. Dobermann
15. Lhasa Apso
16. Miniature Schnauzer
17. Rough Collie
18. Shetland Sheepdog
19. Bulldog
20. Bull Terrier
