# Supplementary figures and images for: Trends in genetic diversity for all Kennel Club registered pedigree dog breeds
Source: Canine Genet Epidemiol. 2015 Sep 21;2:13. doi: 10.1186/s40575-015-0027-4 (PMC4579366; doi:10.1186/s40575-015-0027-4)

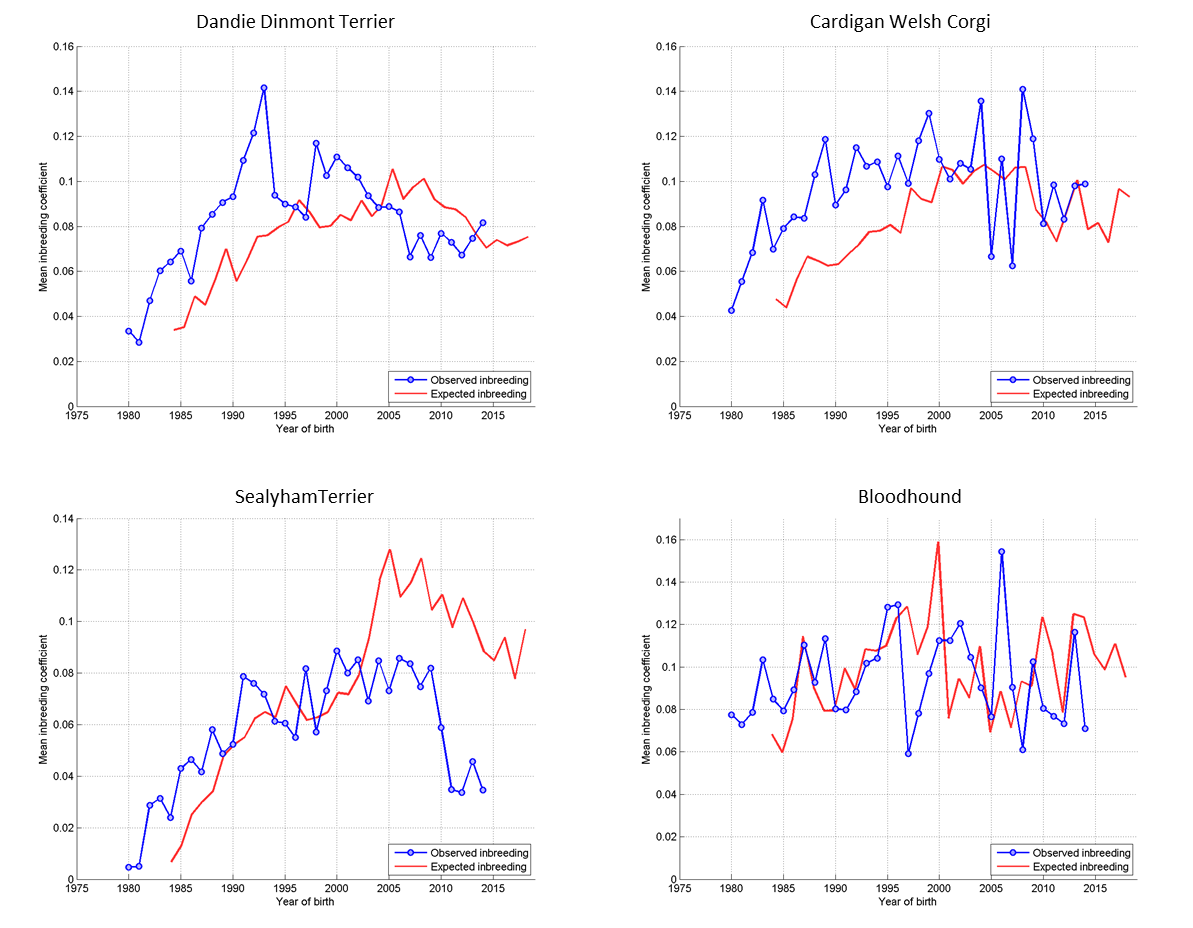

Supplement: Additional file 4: Figure S1. — The plots of observed and expected inbreeding for four breeds showingsmall divergence between the two. (PNG 204 kb) [file 40575_2015_27_MOESM4_ESM.png]

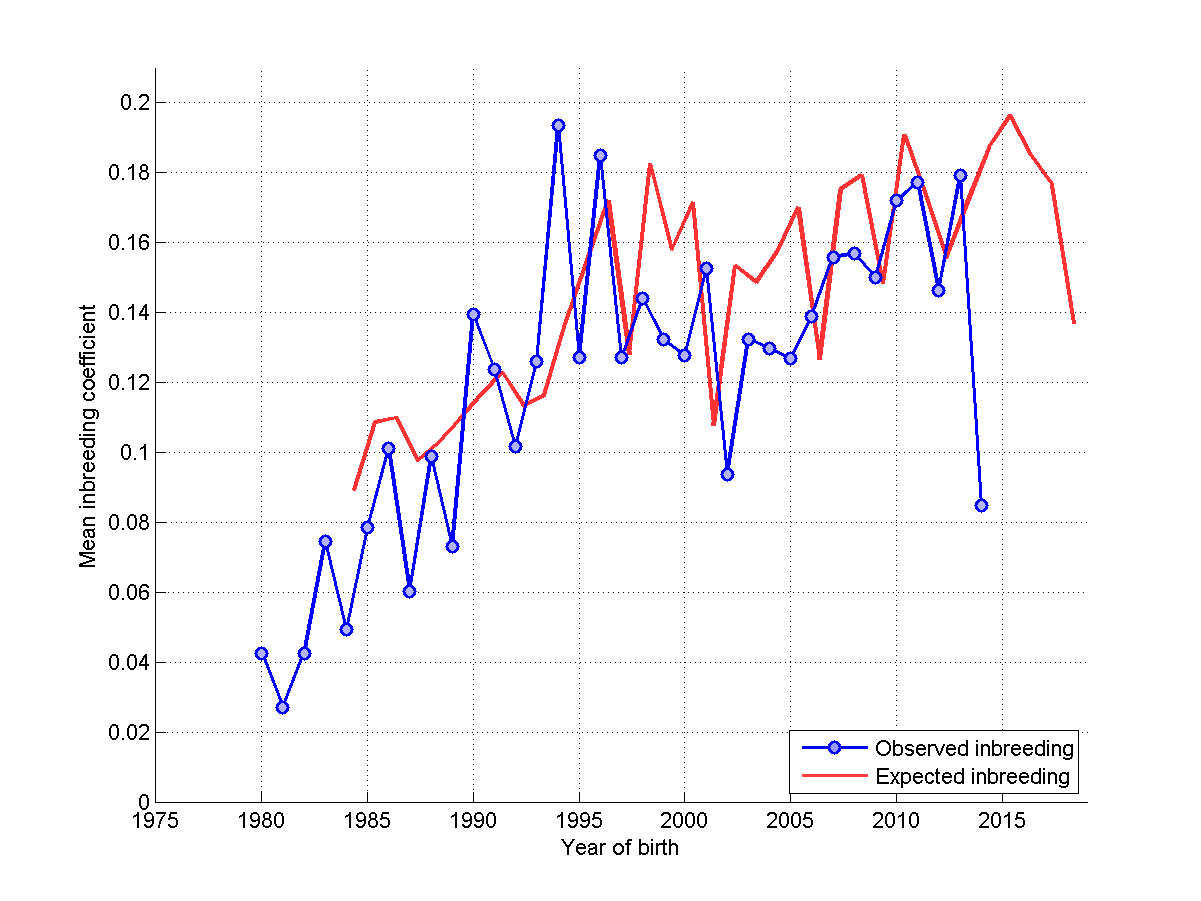

Supplement: Additional file 5: Figure S2. — Plot of observed and expected inbreeding for the Otterhound breed, showingsmall divergence but a rise in both. (PNG 25 kb) [file 40575_2015_27_MOESM5_ESM.png]

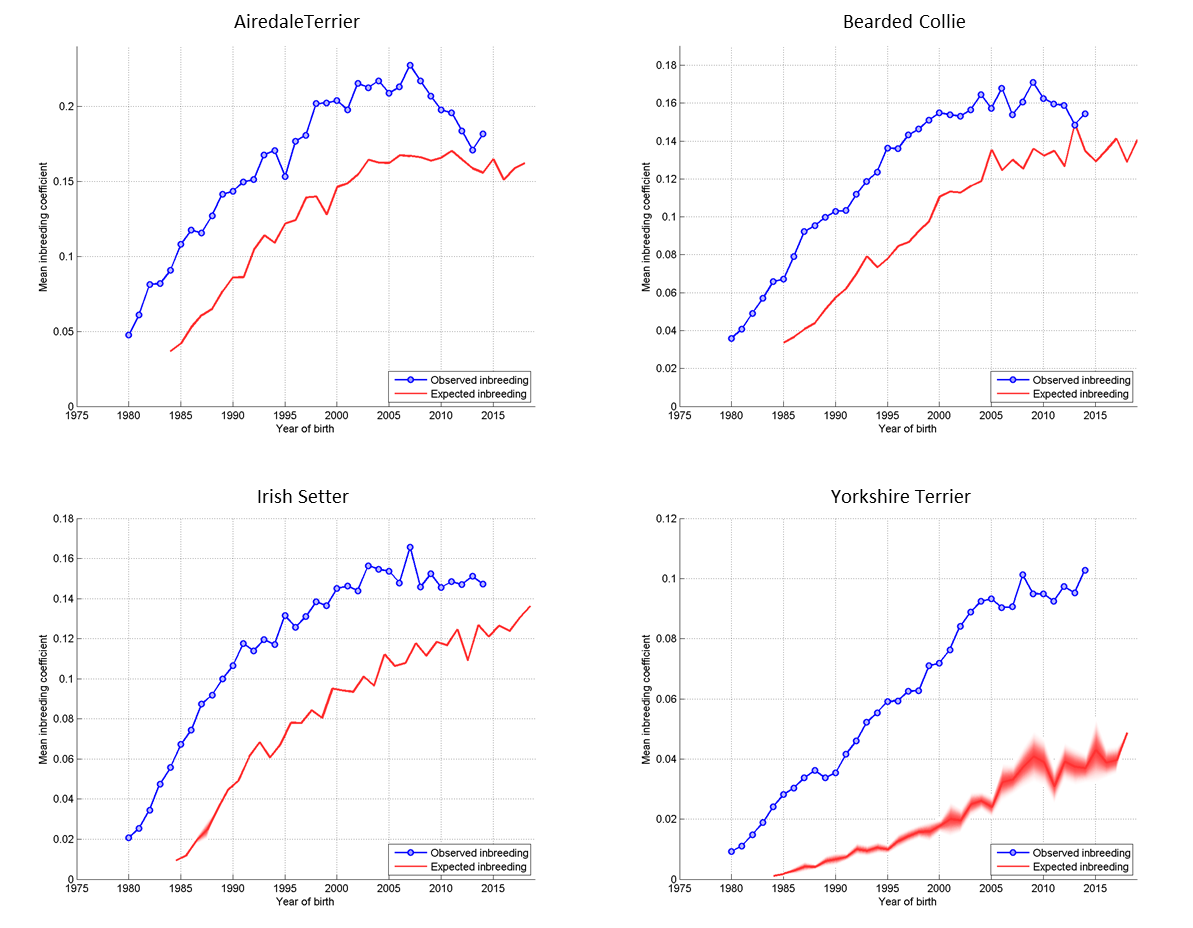

Supplement: Additional file 6: Figure S3. — The plots of observed and expected inbreeding for four breeds showing a steeprise in observed inbreeding, and therefore a low effective population size (Ne). (PNG 183 kb) [file 40575_2015_27_MOESM6_ESM.png]
